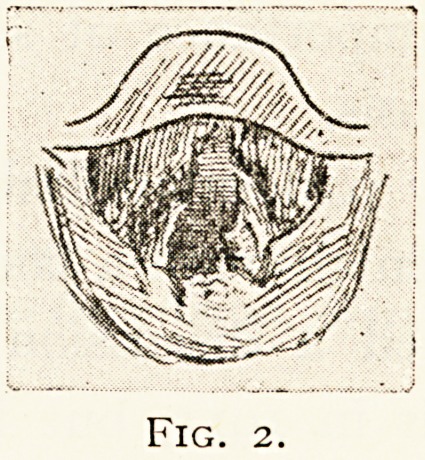# Note on the Treatment of Laryngeal Tuberculosis by Tuberculin

**Published:** 1914-06

**Authors:** P. Watson-Williams

**Affiliations:** Lecturer on Otology, Laryngology and Rhinology, University of Bristol, and in charge of the Ear, Nose and Throat Department, Bristol Royal Infirmary


### NOTE ON THE TREATMENT OF LARYNGEAL TUBERCULOSIS BY TUBERCULIN.

BY

P. Watson-Williams, M.D. Lond.,

*Lecturer on Otology, Laryngology and Rhinology, University of Bristol, and in charge of the Ear, Nose and Throat Department, Bristol Royal Infirmary.*

---

The value of tuberculins in the treatment of tuberculosis has been raised in the course of the discussion introduced by such distinguished authorities as Dr. Habershon and Dr. Noel Bardswell, and it may, therefore, be of interest to summarise very briefly the results which I have experienced with this form of vaccine therapy in relation to laryngeal complications of pulmonary tuberculosis. So exceedingly rare is primary laryngeal tuberculosis, that it is to be regarded as merely a possible pathological curiosity, the existence of which can only be determined by a *post-mortem* examination thus for all practical purposes primary laryngeal tuberculosis is non-existent, and hence we should only consider laryngeal manifestations of tuberculosis as complications of pre-existing, or at any rate co-existing, pulmonary lesions.

Furthermore, I would state at the outset that I have too little faith in tuberculin to rely alone on any varieties of tuberculin in the treatment of a patient, and I consider that, however valuable, it should be used as an adjunct to general hygienic and therapeutic measures, and oftentimes to other methods of local therapy, such as, on the one hand, all that modern sanatorium treatment connotes, and on the other ablation, galvano-cauterisation, submucous injections of guaiacol, and so forth. Nor do I fail to realise very fully the value of complete and absolute abstention from phonation so as to ensure as far as possible laryngeal rest. Indeed, I believe that when speaking at the Annual Meeting of the British Medical Association in 1901 I was the first to insist on the importance of such physiological rest in laryngeal tuberculosis.

Many patients referred to me for laryngeal tuberculosis have been through a course of tuberculin injections since I introduced a discussion on the treatment of tuberculous laryngitis in 1911, but I have seen no reason to alter my practice or my views as to the value of such injections, and I therefore will cite from my remarks on that occasion.

My own experience with the use of tuberculin in laryngeal tuberculosis began in Jan., 1891, when I used Koch's original tuberculin. In the latter part of that year I reported the results obtained in four cases, but they were all advanced cases of pulmonary tuberculosis, with extensive laryngeal disease, and, as might now be expected, the results were not altogether encouraging. The initial dose in each case was 0.001 c.c. of the original fluid, gradually increased to 0.015 c.c. or more. In one case typical tuberculous ulcerations of the vocal cord healed rapidly. He had seven injections in all, gained 9 lb. in weight in his last three weeks in the Royal Infirmary ward, and left very greatly improved and with apparently a normal larynx.

What impressed me most of all in these cases was the very remarkable sedative effect of the injections, and in one case it was so pronounced that I gave the final injection of 0.1 c.c. solely for the purpose of obtaining relief from the intense pain in the larynx. Sprays and inhalations had been unavailing, but the injection was followed by marked relief within a few hours, and did not return during the remaining eight days the patient continued to live. As a matter of historical interest, I reproduce the sketches of the laryngeal conditions in one of these cases treated with Koch's tuberculin twenty-three years ago (Fig. 1, M.H., Jan. 21st, 1891, Fig. 2, M.H., March 11th, 1891), showing marked improvement after injections.

FIG. 1.

FIG. 2.

Since those days we have learnt the importance of early diagnosis, and also the value of small doses, and so for some years it has been my rule to aim at just avoiding any definite febrile reaction. I generally use either bacillary emulsion, beginning with $\frac{1}{5000}$ grm. or P.T.O. in gradually increasing dosage from 0.0001 running up to 1.0 c.c., giving about two injections a week; and then continuing with either bacillary emulsion or else P.T., beginning with 0.02 c.c., and gradually increasing the dose. Having begun with the small dose, one carefully notes the occurrence of any febrile reaction as the dose increases, and when any given injection is followed by a rise of temperature the subsequent dose should be either the same or even a less amount, until no reaction follows; then the gradually increasing scale of dosage is resumed.

Personally I have been impressed with the value of tuberculins used in this way, and although in a successful result and arrest of the disease it may be impossible to precisely apportion the credit due to the tuberculin and the other general measures, the conviction remains that suitable cases treated with tuberculin run a more favourable course in consequence.

Holding such convictions, it would be unjustifiable to withhold any factor which makes for success in dealing with a mortal disease in order to prove to one's satisfaction that this or that factor is the more essential.

## Figures and Tables

**Fig. 1. f1:**
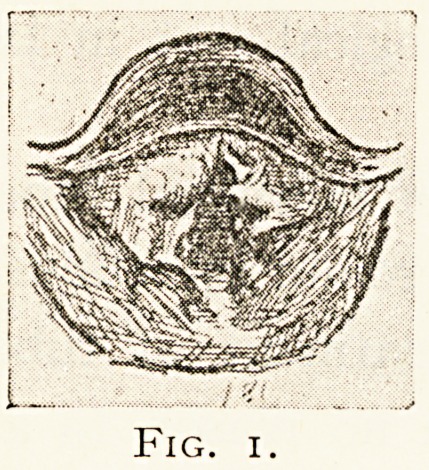


**Fig. 2. f2:**